# Combined deletion of cytosolic 5′-nucleotidases IA and II lowers glycemia by improving skeletal muscle insulin action and lowering hepatic glucose production

**DOI:** 10.1016/j.jbc.2025.108295

**Published:** 2025-02-11

**Authors:** Roxane Jacobs, Gaëtan Herinckx, Noémie Galland, Clémence Balty, Didier Vertommen, Mark H. Rider, Manuel Johanns

**Affiliations:** 1 Université catholique de Louvain (UCLouvain) and de Duve Institute (DDUV), Brussels, Belgium; 2 Université de Lille, Institut Pasteur de Lille and INSERM, Lille, France

**Keywords:** AMP-activated protein kinase (AMPK), glycemic regulation, liver glucose production, muscle glucose uptake, purine nucleotide metabolism

## Abstract

Obesity and type 2 diabetes (T2D)-linked hyperglycemia, along with their associated complications, have reached pandemic proportions, constituting a major public health issue. Genetic deletion or pharmacological inhibition of purine nucleotide-metabolizing enzymes has emerged as a potential strategy for treating diseases. We previously showed that cytosolic 5′-nucleotidase II (NT5C2)-deficient mice were protected against high-fat diet (HFD)-induced insulin resistance. This study investigated the effects of dual deletion of cytosolic 5′-nucleotidases IA (NT5C1A) and II (NT5C2) in mice. We found that NT5C1A/NT5C2 double-knockout (NT5C-dKO) mice exhibited mild hypoglycemia, associated with enhanced skeletal muscle insulin action and reduced hepatic glucose production. This phenotype was accompanied by liver and skeletal muscle proteomic alterations notably related to amino acid metabolism, besides the potential involvement of adenosine monophosphate (AMP)-activated protein kinase (AMPK). Our findings support the development of novel anti-diabetic treatments using small-molecule cytosolic 5′-nucleotidase inhibitors.

Glycemia is determined by a balance between dietary intake, tissue uptake and utilization of glucose, and hepatic gluconeogenesis. After a meal, blood glucose levels rise, triggering insulin release from the pancreas. Insulin stimulates glucose uptake, primarily by skeletal muscle, while inhibiting hepatic glucose production. The net effect is a rapid return of glycemia to normal levels. Type 2 diabetes (T2D) is characterized by insulin resistance stemming from a general loss of insulin sensitivity in cells and tissues. As a result, insulin in T2D fails to restore normal blood glucose levels after meals and to suppress hepatic glucose production, leading to hyperglycemia in both the postprandial and the starved states. AMP-activated protein kinase (AMPK) activation could be beneficial for treating diabetes by stimulating skeletal muscle glucose uptake (1, 2) and perhaps by inhibiting hepatic gluconeogenesis, although the latter is somewhat controversial (3).

We and others have explored targeting enzymes that metabolize purine nucleotides, specifically AMP and inosine 5′-monophosphate (IMP), as a potential strategy for AMPK activation *in vivo* (4, 5, 6, 7, 8, 9). In most tissues, AMP is either directly hydrolyzed to adenosine by cytosolic 5′-nucleotidase IA (NT5C1A) or converted to IMP by AMP deaminase (AMPD). IMP can then be hydrolyzed to inosine by cytosolic 5′-nucleotidase II (NT5C2) (Fig. 1*A*). NT5C1A expression is high in skeletal muscle but is low in liver, whereas NT5C2 is widely expressed, including in liver (Fig. 1, *B* and *C*).

**Figure 1:**
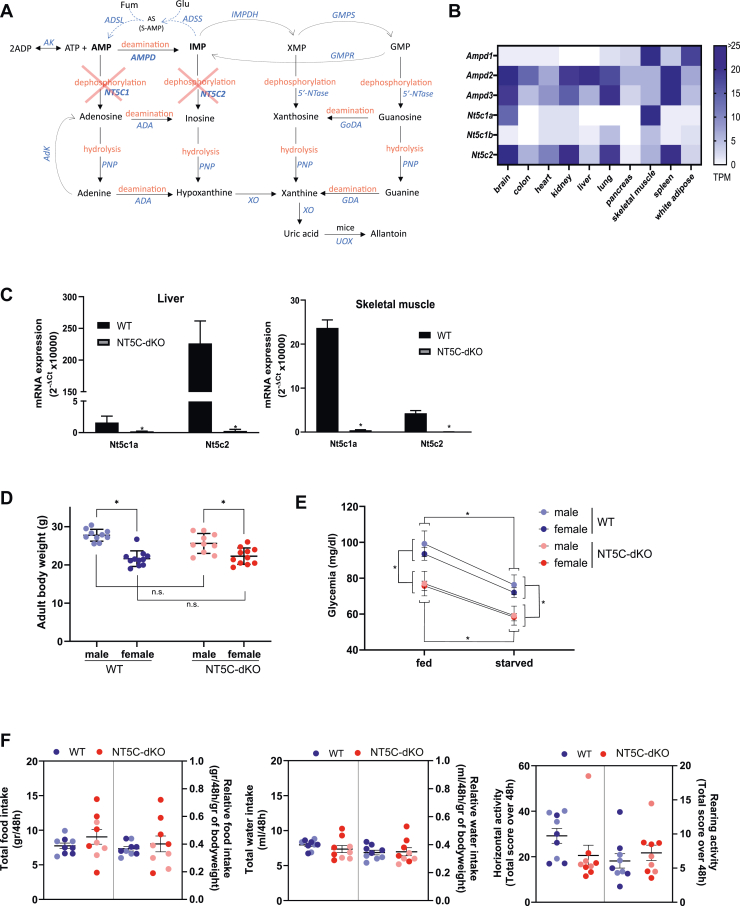
**NT5C-dKO mice are mildly hypoglycemic.** Purine nucleotide metabolism involves conversion of AMP to IMP by AMPDs and dephosphorylation of AMP to adenosine by NT5C1 or IMP to inosine by NT5C2 (*A*). IMP can be converted back to AMP *via* AS or directed towards the XMP/GMP pathways. AMP/IMP-metabolizing enzymes have distinct tissue distribution patterns. Data are for mouse tissues and were retrieved from the European Bioinformatics Institute (EBI) Expression Atlas (*B*). NT5C1A and NT5C2 mRNA levels were measured by RT-qPCR in skeletal muscle and liver from WT and NT5C-dKO mice (n = 5 per genotype) to confirm deletion of the two enzymes (*C*). Adult male (n = 10 per genotype) and female (n = 11 per genotype) WT and NT5C-dKO mice were weighed, data were analyzed by two-way ANOVA with Sidak’s *post hoc* test and ∗ indicates a significant (*p* < 0.05) difference between indicated groups, while “n.s.” indicates no significant difference (*D*). Glycemia was measured on blood drops from the tail of fed and overnight starved male (n = 6 per genotype) and female (n = 7 per genotype) WT and NT5C-dKO mice, data were analyzed by three-way ANOVA with Tukey’s *post hoc* test and ∗ indicates a significant (*p* < 0.05) difference between indicated groups; no significant difference was seen between sexes regarding the effects of the genotype and the feeding state (*E*). WT and NT5C-dKO male (n = 4 per genotype, clear symbols) and female (n = 5 per genotype, dark symbols) mice were monitored for 48 h in physio cages for measurements of food consumption (*left panel*), water consumption (*middle panel*) and locomotor activity (*right panel*); data were analyzed by an unpaired *t* test with Welch’s correction for unequal variances; as no significant difference was seen between sexes, males and females were analyzed together (*F*).

In mice deficient for AMPD1, the major isoenzyme in skeletal muscle, *ex vivo* muscle contraction led to increased AMP levels, but this did not enhance glucose uptake (4). Also, genetic deletion or pharmacological inhibition of AMPD1 failed to improve glycemic control in insulin-resistant HFD-fed mice (9). Conversely, deletion of the major liver isoenzyme AMPD2 had beneficial effects in mice fed a high-fructose diet, primarily by reducing hepatic glucose production (8).

Single whole-body knockout of either NT5C1A or NT5C2 did not lead to increased AMPK activation or contraction-induced glucose uptake in incubated skeletal muscles from chow-fed mice (6). Our studies were at variance with those of others who showed that knockdown of NT5C1A or NT5C2 led to modestly increased skeletal muscle AMPKα Thr172 phosphorylation and glucose uptake (10). Also, NT5C2 silencing was shown to activate AMPK in lung tumor cells (11). However, we found that NT5C2-deficient mice were protected from HFD-induced weight gain, adiposity, insulin resistance, and hyperglycemia (7), with a possible implication of AMPK. Building on these studies, we generated the first *in vivo* model for dual deletion of NT5C1A and NT5C2 (referred to as “NT5C-dKO” mice) and investigated effects on glycemic control, hepatic glucose production, insulin action in skeletal muscle, and muscle/liver protein expression.

## Results

### Mice lacking both NT5C2 and NT5C1A have a mild hypoglycemic phenotype

Due to the lack of adequate antibodies for detecting NT5C1A and NT5C2 in tissue extracts, their expression was assessed at the mRNA level by qPCR. Expression of both Nt5c2 and Nt5c1a mRNA was quasi-totally absent in skeletal muscle and liver from NT5C-dKO *versus* WT mice (Fig. 1*C*), indicating efficient deletion of both nucleotidases. There was no apparent phenotypic difference in male or female NT5C-dKO compared to WT mice, as body weight, food/water intake and locomotor activity were similar (Fig. 1, *D* and *F*). However, glycemia was significantly lower in NT5C-dKO mice both in the fed state and after overnight starvation (Fig. 1*E*), suggesting alterations in glycemic control.

### NT5C-dKO mice have improved glucose clearance through enhanced skeletal muscle insulin action

**Figure 2:**
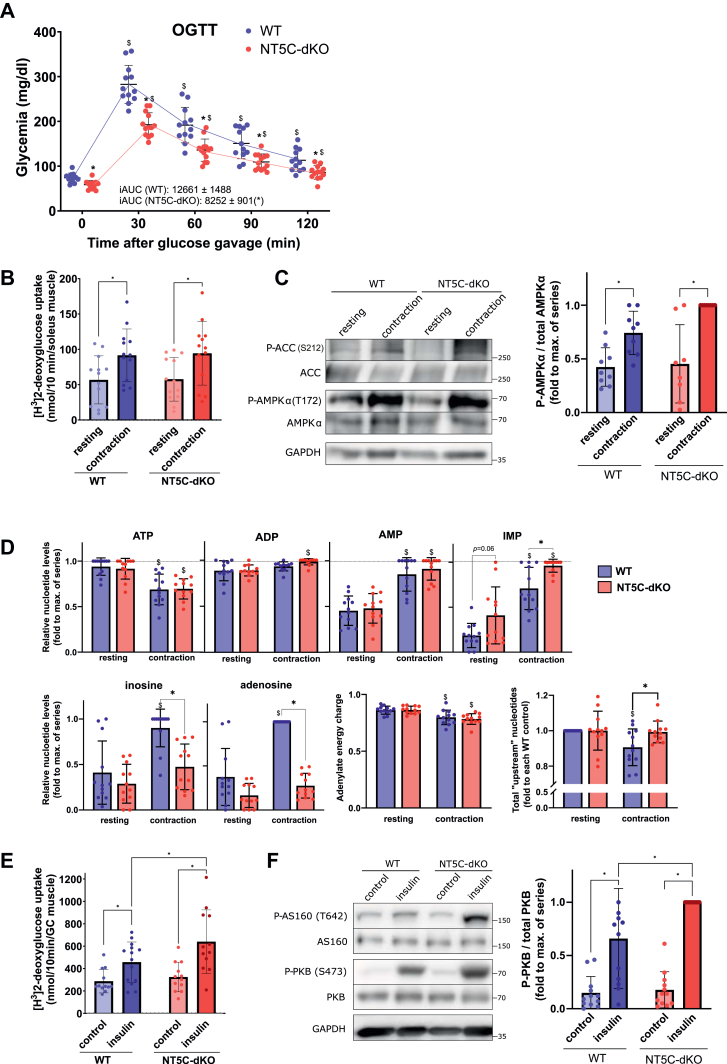
**Insulin-stimulated glucose uptake is increased in muscles from NT5C-dKO mice.** Starved WT (n = 12, 6 males and 6 females) and NT5C-dKO mice (n = 13, 6 males and 7 females) were subjected to an oral glucose tolerance test (2 mg glucose/g of body weight) and glycemia was measured on blood drops from the tail at the indicated times over 2 h (*A*). iAUC is the incremental area under the curve using the starting (0 min) value of each group used as baseline. Means ± SD are displayed along with individual values. Data were analyzed using two-way ANOVA for repeated measurements with Tukey’s *post hoc* test, ∗ indicates a significant (*p* < 0.05) difference between genotypes (WT as reference) and $ between time points (0 min as reference); as no significant difference was seen between sexes, males and females were analyzed together. Isolated soleus muscles from WT (n = 12, 6 males and 6 females) and NT5C-dKO (n = 13, 6 males and 7 females) mice were incubated *ex vivo* for 30 min, either with or without electrical stimulation (contraction) (*B*–*D*) or in the presence or absence of 100 nM insulin (*E*, *F*) for measurement of [^3^H]-2-deoxyglucose uptake during an additional 10 min of incubation (*B*, *E*), immunoblotting with the indicated antibodies and densitometric band quantification (*C*, *F*) or measurement of purine nucleotides in perchloric acid extracts by HPLC (*D*). In (*D*), “upstream” nucleotides are ATP + ADP + AMP + IMP. The adenylate energy charge is defined as [(ATP) + 0.5(ADP)]/[(ATP) + (ADP) + (AMP)]. Data were analyzed by two-way ANOVA with Tukey’s *post hoc* test and ∗ indicates a significant (*p* < 0.05) difference between indicated groups; as no significant difference was seen between sexes, males and females were analyzed together.

When subjected to an oral glucose tolerance test, blood glucose concentrations at all time points were significantly lower in NT5C-dKO compared to WT mice, and the area under the curve (AUC) over 2h following glucose gavage was significantly reduced in NT5C-dKO mice (Fig. 2*A*).

As glucose removal primarily occurs *via* uptake into skeletal muscle, which is stimulated both by contraction and insulin, we looked whether this process was altered in NT5C-dKO mice. *Ex vivo* contraction of soleus muscles resulted in increased [^3^H]-2-deoxyglucose uptake as expected (Fig. 2*B*) but was similar in muscles from WT and NT5C-dKO mice. Measurements of intramuscular purine nucleotide concentrations indicated that contraction-induced increases in AMP were similar in soleus muscles from WT *versus* NT5C-dKO mice, whereas the rise in IMP was potentiated (Fig. 2*D*). Immunoblotting of muscle extracts indicated similar contraction-induced increases in phosphorylation of AMPKα Thr172 and P-ACC S212 (Fig. 2*C*), a direct AMPK target, suggesting there was no difference in AMPK activation due to contraction in skeletal muscle from WT *versus* NT5C-dKO mice.

By contrast, insulin-stimulated [^3^H]-2-deoxyglucose uptake was significantly enhanced in *ex vivo* incubations of gastrocnemius muscles from NT5C-dKO *versus* WT mice (Fig. 2*E*). Accordingly, insulin-induced PKB Ser473 phosphorylation was potentiated in extracts of muscles from NT5C-dKO mice compared to WT mice (Fig. 2*F*), suggesting improved muscle insulin sensitivity due to dual deletion of NT5C1A and NT5C2. Therefore, an intraperitoneal insulin tolerance test on WT *versus* NT5C-dKO mice was undertaken. Using a submaximal dose of insulin, glycemia was significantly reduced in NT5C-dKO *versus* WT mice (Fig. S1*A*). In addition to improved systemic insulin responsiveness, plasma insulin following re-feeding was increased in NT5C-dKO compared with WT mice (Fig. S1*B*), which could not be explained by increased pancreatic insulin content (Fig. S1*C*), suggesting improved pancreatic insulin release in NT5C-dKO mice in response to a feeding-induced rise in blood glucose.

### Dual NT5C1A/NT5C2 deletion lowers hepatic glucose production

**Figure 3:**
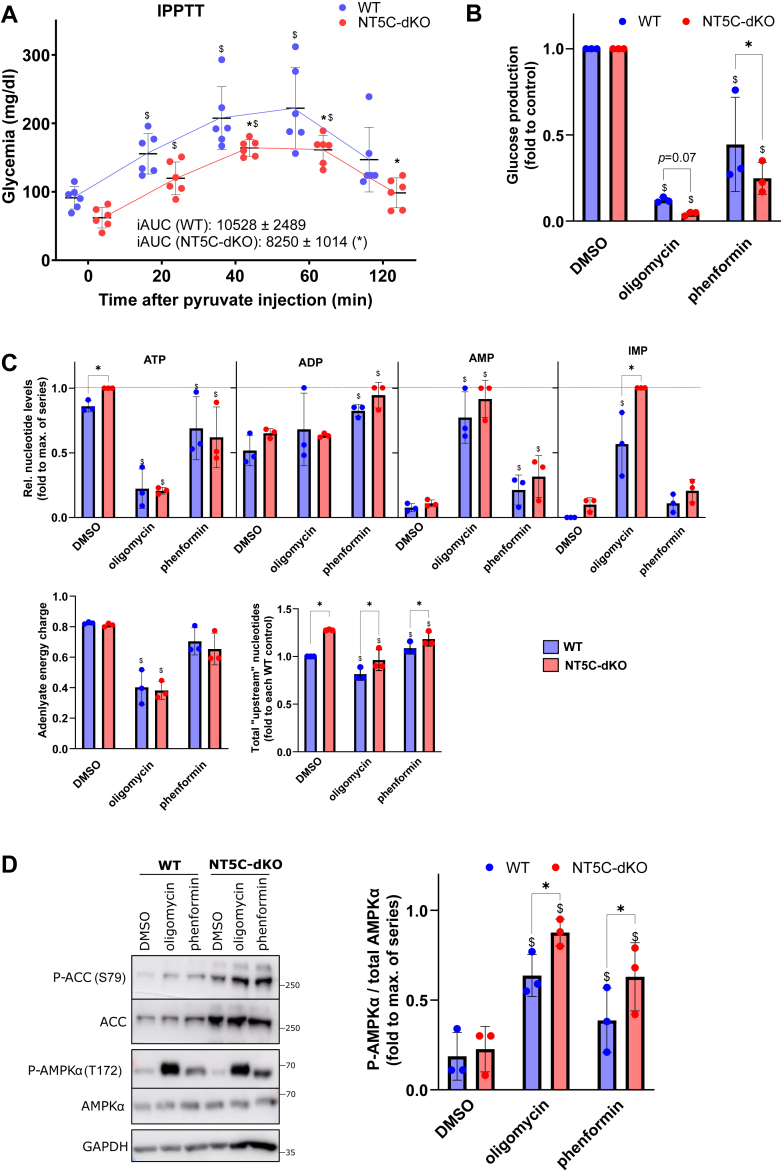
**NT5C-dKO mice display decreased hepatic glucose production.** Starved WT (n = 6, 3 males and 3 females) and NT5C-dKO mice (n = 6, 3 males and 3 females) were subjected to an intraperitoneal pyruvate tolerance test (1 mg pyruvate/g of body weight) and glycemia was measured on blood drops from the tail at the indicated intervals over 2 hours (*A*). iAUC is the incremental area under the curve using the starting (0 min) value of each group used as baseline. Means ± SD are displayed along with individual values. Data were analyzed using two-way ANOVA for repeated measurements with Tukey’s *post hoc* test, ∗ indicates a significant (*p* < 0.05) difference between genotypes (WT as reference) and $ between time points (0 min as reference); as no significant difference was seen between sexes, males and females were analyzed together. Hepatocytes were isolated from the livers of WT and NT5C-dKO mice (n = 3 per genotypes, males), cultured overnight and incubated for 2 h with or without 1 μM oligomycin or 0.5 mM phenformin, either in glucose-free media supplemented with 10 mM lactate and 1 mM pyruvate for enzymatic determination of newly produced glucose in the media (*B*), or in normal media containing glucose for immunoblotting with the indicated antibodies and densitometric band quantification (*C*) or measurement of purine nucleotides in perchloric acid extracts by HPLC (*D*). In (*D*), nucleotides “upstream” of NT5C1A/2 are ATP + ADP + AMP + IMP. The adenylate energy charge is defined as [(ATP) + 0.5(ADP)]/[(ATP) + (ADP) + (AMP)]. Data were analyzed by two-way ANOVA with Tukey’s *post hoc* test and ∗ indicates a significant (*p* < 0.05) difference between genotypes compared to WT and ^$^ between treatments compared to DMSO vehicle control.

As hepatic glucose production is the main determinant of glycemia during starvation, we looked whether the observed differences in blood sugar after starvation could be due to reduced glucose synthesis. This was indeed suggested by an intraperitoneal pyruvate tolerance test, during which the rise in glycemia was significantly lower in NT5C-dKO compared to WT mice (Fig. 3*A*). Accordingly, the metabolic stress-induced reduction in glucose production was more pronounced in hepatocytes from NT5C-dKO compared to WT mice (Fig. 3*B*). In these experiments, metabolic poisons were used to increase the flux through the purine nucleotide system, namely phenformin, which inhibits mitochondrial complex I or oligomycin, which inhibits mitochondrial ATP synthase. This results in decreased mitochondrial ATP production and a concomitant increase in AMP levels. AMPKα Thr172 phosphorylation was slightly higher in NT5C-dKO *versus* WT hepatocytes incubated with both compounds (Fig. 3*C*). In fact, physiological energy stress is encountered in the liver during long-term starvation (12), accompanied by a drop in ATP and a rise in AMP levels. In hepatocytes, AMP and IMP are potent negative regulators of glucose production, by inhibiting fructose-1,6-bisphophatase (FBPase-1) catalyzing a key step of gluconeogenesis (13). Interestingly, levels of IMP but not of AMP were significantly enhanced in hepatocytes from NT5C-dKO *versus* WT mice incubated with oligomycin or phenformin (Fig. 3*D*), which might have been due to rapid conversion of AMP to IMP by AMPD. Under these conditions, nucleotides can be rerouted toward the TCA cycle through a salvage pathway involving conversion of IMP plus glutamate to adenylosuccinate (AS) and fumarate by AS synthase (ADSS) and back to AMP by AS lyase (ADSL) (Fig. 1*A*). Indeed, by GC-MS, glutamate levels were decreased in NT5C-dKO *versus* WT hepatocytes, while fumarate and malate were increased in presence of phenformin (Fig. S2).

### Muscle and liver proteomes are remodeled in NT5C-dKO mice

**Figure 4:**
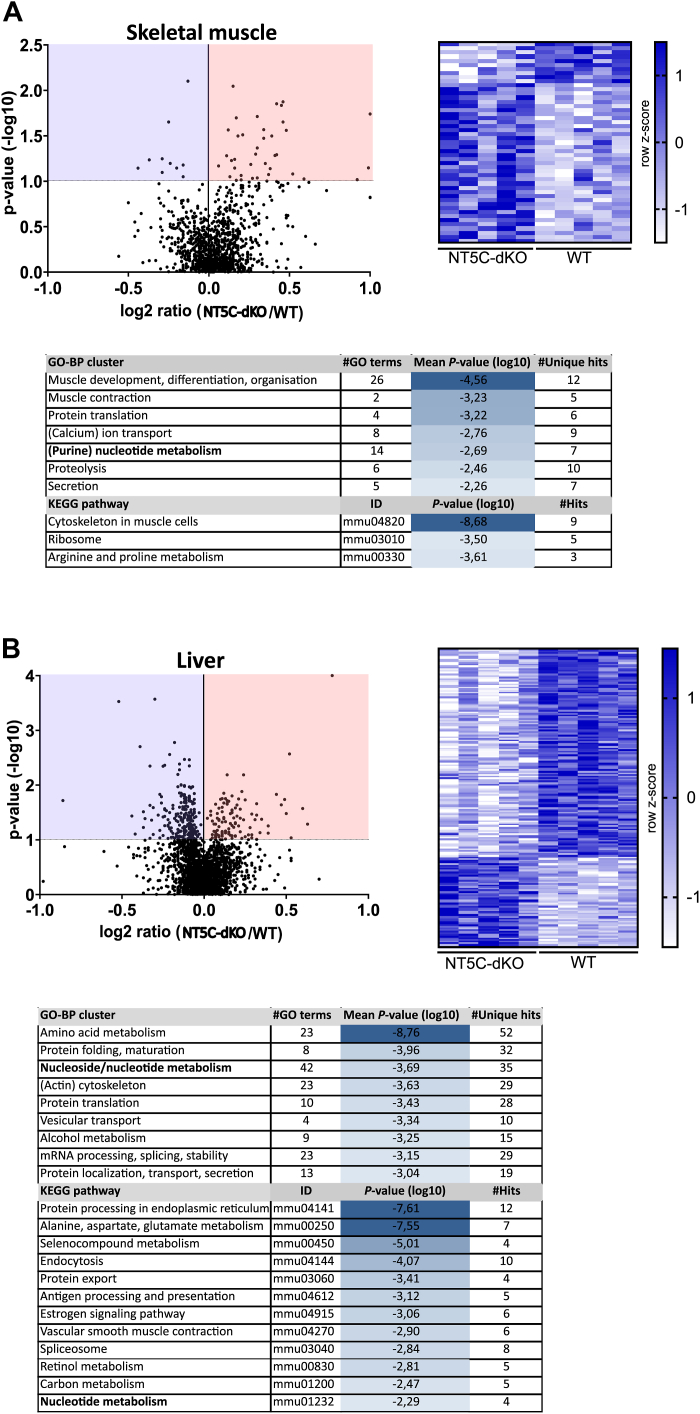
**Proteomic remodeling in muscle and liver of NT5C-dKO mice.** Tibialis anterior (TA) muscles (*A*) and livers (*B*) from WT and NT5C-dKO mice (n = 5 per genotype, 3 males and 2 females) were collected for protein extraction, trypsin digestion, TMT peptide labelling and proteomic analysis by LC-MS/MS. Differentially abundant proteins (FDR < 0.1 using the Benjamini-Hochberg correction) are highlighted in volcano plots and represented in heatmaps for each tissue, along with functional enrichment analysis for manually renamed GO-BP clusters and KEGG pathways The lists of proteins identified along with the results of the statistical analysis and details of the enrichment can be found in Table S1.

To investigate possible mechanisms underlying the changes in the regulation of skeletal muscle glucose uptake and liver glucose production, we analyzed the proteome of both tissues by LC-MS/MS. Using TMT labeling, we were able to robustly quantify over 3000 proteins (Fig. 4, *A* and *B*). Differences in abundance of 49 and 181 proteins (*P*_adj_.<0.1) were seen in NT5C-dKO *versus* WT muscles and livers, respectively (Fig. 4, *A* and *B*, and Table S1). Interestingly, AS synthase was among the proteins slightly more abundant in NT5C-dKO *versus* WT livers. Functional analysis of associated GO-BP and KEGG terms revealed that these differential proteins were enriched for nucleotide metabolism, as expected, but also strongly for amino acid metabolism (Fig. 4, *A* and *B*). Indeed, (purine) nucleotide and amino acid metabolism have multiple connections at the level of *de novo* synthesis, catabolism, and salvage pathways (14). Together, these data indicate that NT5C-dKO mice undergo a subtle proteomic remodeling, possibly to reroute nucleotide catabolism.

### Pharmacological NT5C2/NT5C1 inhibition decreases hepatocyte glucose production

**Figure 5:**
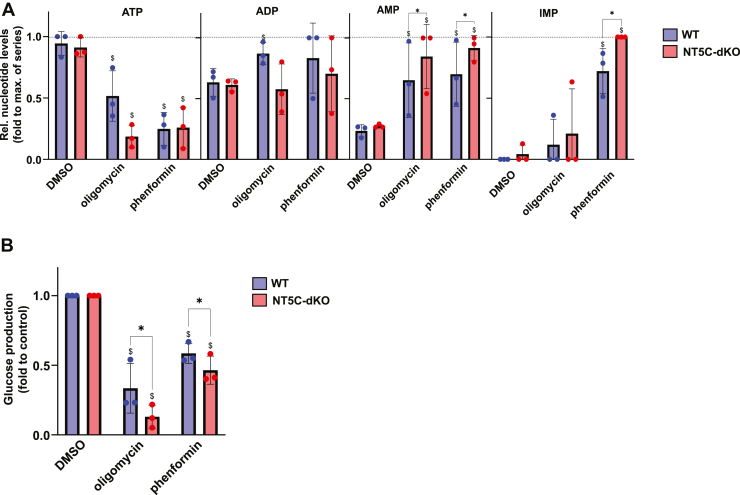
**Pharmacological inhibition of NT5C1 and NT5C2 in hepatocytes recapitulates genetic deletion.** Hepatocytes were isolated from the livers of WT and NT5C-dKO mice (n = 3 per genotype, males), cultured overnight and incubated for 4 h in glucose-free media supplemented with 10 mM lactate and 1 mM pyruvate with or without 0.5 μM oligomycin or 0.5 mM phenformin, alone or in combination with 0.5 mM EdU and 10 μM CRCD2 to inhibit NT5C1 and NT5C2, respectively, for measurement of purine nucleotides in perchloric acid cell extracts by HPLC (*A*) or enzymatic determination of glucose in incubation media (*B*). Data were analyzed by two-way ANOVA with Tukey’s *post hoc* test and ∗ indicates a significant (*p* < 0.05) difference between genotypes compared to WT and ^$^ between treatments compared to DMSO vehicle control.

To explore the therapeutic potential of our findings, we attempted to pharmacologically mimic the dual deletion of NT5C1A and NT5C2, by the combined use of inhibitors on cells/tissues from WT mice. The nucleotide analog 5-ethynyl-2′-deoxyuridine (EdU) has been used to inhibit NT5C1A (15), while the recently developed small-molecule inhibitor “CRCD2” inhibits NT5C2 (16). In *ex vivo* incubated skeletal muscles, both basal and contraction-induced glucose uptake, and nucleotide levels were unaltered by incubation with both inhibitors (data not shown). We therefore tested their effect with longer incubation times in primary hepatocytes, a more flexible model. When metabolic stress was induced by oligomycin or phenformin treatment, AMP and IMP levels increased to higher levels in the presence of NT5C inhibitors (Fig. 5*A*). Accordingly, the phenformin and oligomycin-induced inhibition of glucose production was stronger in the presence of NT5C inhibitors (Fig. 5*B*).

## Discussion

T2D-linked hyperglycemia is a major global public health issue and predisposes affected individuals to metabolic and cardiovascular disease. Existing treatments have the downside of causing adverse side-effects and the potential development of resistance and/or intolerance. Increased cellular purine dephosphorylation is a feature seen in both type 1 and type 2 diabetic patients (17), while plasma hypoxanthine (18), uric acid (19), xanthine and adenosine (20) were positively correlated to the incidence of T2D. In the present study, we therefore propose an alternative strategy to lower glycemia, by dual inhibition of soluble 5′-nucleotidases (NT5Cs). Overall, we found that purine nucleotide metabolism was remarkably resilient towards metabolic stress or genetic manipulation, potentially because fluxes through these enzymes are low and because, from IMP and glutamate, adenine nucleotides can be rerouted towards the TCA cycle *via* ADSS/ADSL-mediated fumarate production (Fig. 1*A*), with other possible connections to amino acid metabolism through the TCA cycle. In addition, nucleotides and derivates can be exported to the extracellular space (21, 22, 23), which might also explain why intracellular AMP and IMP did not further accumulate in our model.

Nevertheless, NT5C-dKO mice displayed mild hypoglycemia and improved glucose clearance *via* 1) enhanced skeletal muscle insulin action, 2) lower hepatic glucose production (in the presence of metabolic stress), and 3) increased feeding-induced pancreatic insulin release. We were able to link this phenotype to proteomic changes in skeletal muscle and liver, mainly associated with amino acid metabolism (Fig. 4). The full complexity of enhanced insulin secretion and signaling by NT5C deletion remains to be explored but is probably multifaceted involving different organs. Purine metabolism was shown to be implicated in the control of glucose metabolism, including insulin release and action (24, 25, 26). High plasma uric acid directly inhibits insulin signaling inducing insulin resistance (27), and we showed that NT5C2 deletion in mice lowered HFD-induced hyperuricemia (7). Interestingly, AS has been described as a pancreatic insulin secretagogue (28), which might be a feature participating in the improved glucose clearance and enhanced feeding-induced insulin secretion of NT5C-dKO mice. Although potentiation of stress-induced levels of intracellular AMP and AMPKα Thr172 phosphorylation were modest at best, we cannot rule out a role for AMPK activation in the hypoglycemic phenotype seen upon dual NT5C1A-NT5C2 deletion. AMPK activation *in vivo* is difficult to study because once tissues go anoxic during dissection prior to freeze-clamping, AMPK becomes activated. Of interest in explaining effects seen in NT5C-dKO mice is the enhanced rise in IMP seen during muscle contraction (Fig. 2*D*) and in hepatocytes incubated with mitochondrial poisons (Fig. 5*A*). We estimate that the maximal concentrations that would be reached in skeletal muscle and hepatocytes would be around 500 nmol/g of wet weight and 5 nmol/10^6^ cells, respectively. Although IMP does not appear to allosterically stimulate AMPK activity nor protect against AMPKα Thr172 dephosphorylation (29), IMP is an allosteric inhibitor of FBPase-1 (13), which might participate in enhanced suppression of hepatic glucose production under metabolic stress (Fig. 5*B*). Another enzyme that is IMP-sensitive is the phosphomannomutase isoenzyme (PMM1) that hydrolyses glucose-1,6-bisphosphate (30), raising the intriguing possibility that reduced glucose-1,6-bisphosphate levels might be linked to the control of glycemia. It is perhaps noteworthy that inhibition of IMP dehydrogenase reduces diet-induced obesity (31). Lastly, dual NT5C inhibition might increase flux through the XMP pathway (Fig. 1*A*), and hypoxanthine supplementation was recently shown to decrease glycemia by suppressing hepatic gluconeogenesis (32).

Our findings support the development of new anti-diabetic compounds based on small-molecule inhibition of cytosolic NT5Cs. In fact, NT5C(2) inhibitors are already being tested in anti-cancer therapy (16) and might be repurposed to treat T2D. Despite the absence of any apparent adverse health effects of whole-body deletion of both NT5C1A and NTC2 in our mouse model, the presence of auto-antibodies against NT5C1A was linked to (dermato)myositis (33) while its overexpression in pancreatic cancer was linked to chemotherapy resistance (34). Total loss by homozygous exon deletion of NT5C2 was associated with spastic paraplegia (35), while gain-of-function mutations in NT5C2 were linked to persistence and chemotherapy resistance of acute lymphoid leukemia (ALL) (16, 36, 37) and NT5C2 expression in breast cancer cells was crucial for resistance to glucose deprivation (38). For long-term treatments, partial inhibition of NT5Cs is likely to be beneficial without major side-effects. Very encouragingly, the first-in-class small molecule NT5C2 inhibitor CRCD2 was efficient against ALL tumor cells both *in vitro* and *in vivo* in mice, without detectable adverse effects (besides minimal weight loss) (16). Pharmacological NT5C2 inhibition was thus safe in mice and should now be tested in humans. To our knowledge, there are currently no small-molecule inhibitors of NT5C1A, and specific pharmacologic inhibition of this enzyme has not yet been attempted *in vivo*. There is thus an increasing need for the development of novel, isoform-specific small-molecule inhibitors of NT5Cs. One option to achieve this could be to target functionally essential enzyme oligomerization, a strategy that was successfully employed by our colleagues to disrupt LDH tetramers required by cancer cells (39), and both NT5C1A and NT5C2 are active as homotetramers (40).

## Experimental procedures

### Materials

Unless otherwise stated, all chemicals were from Merck/Sigma Aldrich. Radiochemicals were from PerkinElmer. Antibodies were from the sources cited. Oligonucleotides were from Integrated DNA Technologies. Compound “CRCD2” was from Enamine Ltd (Ukraine, cat. no. 27358589).

### Animals

A whole-body NT5C1A:NT5C2 double-knockout C57Bl/6N mouse strain was obtained by crossing animals with single whole-body deletions of the two enzymes originally generated by AstraZeneca (Mölndal, Sweden) as previously described (6). Homozygous animals were bred separately from the wild-type (WT) strain for up to three generations. NT5C-dKO mice exhibited no obvious phenotypic differences compared with WT mice and breeding was normal. Mice were housed with a 12 h light-dark cycle and fed *ad libitum* with a standard chow diet and water. Two- to eight-month-old males and females were used for experimentation and compared to age-matched WT animals. No obvious sex-based differences were observed comparing NT5C-dKO and WT mice. At the beginning of *in vivo* experiments, animals were weighed for normalization purposes. All experiments were approved by the Animal Ethics Committee of UCLouvain under reference number 2021/UCL/MD/028 and conducted in accordance with EU Directive 2010/63/EU for animal experimentation.

### Bodyweight, food/water intake, and locomotor activity

After a 1-week adaptation period to re-housing, animals were kept for 72 h in metabolic cages (Bioseb physiocage 00) for measurement of food/water consumption and horizontal movement over the last 48 h at the UCLouvain Animal Behaviour Analysis Platform (BeAP). Animals were weighed at the start of the experiment for normalization.

### RNA extraction and RT-qPCR

Total RNA was extracted, mRNA was retrotranscribed to cDNA using poly-dT primers, and tissue expression of Nt5c1a and Nt5c2 was analyzed as previously described (7) using the following primers (Nt5c1a: forward CTCAGGTGGGAGTTCGTCTCA, reverse GGTAGCAGATGGGGCTATTCC; Nt5c2: forward TGACCGCTTACAGAATGCAG, reverse CGGCTAGGGTATAATCCATATCA) along with Rpl19 (forward GAAGGTCAAAGGGAATGTGTTCA, reverse CCTTGTCTGCCTTCAGCTTGT) as reference.

### Glucose, pyruvate, and insulin tolerance tests

Overnight starved animals were subjected to an oral glucose tolerance test by gavage of 2 mg glucose/g of bodyweight, an intraperitoneal pyruvate tolerance test by injection of 1 mg sodium pyruvate/g of bodyweight, or an insulin tolerance test by intraperitoneal injection of 0.1U of recombinant human insulin (Actrapid, Novo Nordisk). Glycemia was measured on blood drops taken from the tip of the tail at the indicated time intervals over 2 h using a glucometer (Free Style Lite, Abbott). The incremental area under the curve (iAUC) was calculated using the starting baseline of each group.

### Collection of organs and plasma

*Ad libitum* fed and overnight starved mice were euthanized to rapidly collect organs for subsequent analyses. Around 0.5 ml of blood was drawn by cardiac puncture and centrifuged (2000*g* × 10 min at 4 °C) to obtain plasma. The left lobe of the liver was immediately freeze-clamped to avoid ischemia. Skeletal muscles (gastrocnemius and/or soleus) were dissected for *ex vivo* incubation or frozen immediately for subsequent analyses.

### Incubation of skeletal muscles for measurements of glucose uptake and purine nucleotides or immunoblotting

Isolated soleus and gastrocnemius muscles from WT and NT5-dKO mice were incubated in parallel for 30 min with or without electrical stimulation or incubation with 100 nM insulin, respectively, for measurements of [^3^H]-2-deoxyglucose uptake and purine nucleotides in perchloric acid extracts (soleus only), or for protein extraction and immunoblotting as described (7). Nucleotides levels are presented relative to the maximum of each series (muscles from one WT and one NT5C-dKO mouse incubated in parallel); absolute values in nmol/mg of wet weight of maximal group averages were as follows: ATP (1.34), ADP (0.36), AMP (0.11), IMP (0.37), inosine (0.14), adenosine (0.06).

### Incubation of primary hepatocytes for measurements of glucose production and purine nucleotides or immunoblotting

Primary hepatocytes from WT and NT5C-dKO mice were isolated in parallel as described (41). After plate attachment and overnight incubation, cells were either incubated for 2 h with 10 mM lactate plus 1 mM pyruvate in glucose-free media for measurements of glucose production, or in normal media containing glucose, for immunoblotting and measurement of purine nucleotides in perchloric acid extracts and metabolites by GC-MS as described (41, 42). Where indicated, 10 μM N-(3-carbamoyl-4,5,6,7-tetrahydrobenzo-[b]thiophen-2-yl)-1H-benzo[d]imidazole-5-carboxamide (“CRCD2”) (16) and 0.5 mM 5-Ethynyl-2-deoxyuridine (EdU) were included in the incubations to inhibit NT5C2 and NT5C1A, respectively. Nucleotides levels are presented relative to the maximum of each series (cells from one WT and one NT5C-dKO mouse incubated in parallel); absolute values in nmol/10^6^ cells of maximal group averages were as follows: ATP (18.4), ADP (7.9), AMP (7.0), IMP (2.2), inosine (0.7), adenosine (1.62).

### Pancreatic insulin release and content

Before (30 min) and after (30 min) administration of an oral bolus of glucose, blood was harvested for subsequent measurement of plasma insulin by ELISA (Crystal Chem, #90080) following the manufacturer’s instructions. For pancreatic insulin content, pancreata were collected from starved mice and processed as described (43) for ELISA measurement of insulin using the same kit.

### Liver and skeletal muscle proteomes

Whole tibialis anterior muscles or minced liver (∼100 mg) were homogenized in 0.3 ml of lysis buffer containing 50 mM triethylammonium bicarbonate pH 8.5, 0.5% (w/v) sodium deoxycholate, 1% (v/v) Igepal, 0.1% (w/v) SDS, 0.2% (w/v) dodecyl maltoside, 1 mM dithiothreitol, 1 mM EDTA, 0.5 mM phenylmethylsulfonyl fluoride. Extracts were centrifuged (10 min x 20,000*g* at 4 °C) and protein content was estimated by BCA assay (Thermo Scientific). Samples of muscle protein (100 μg) or liver protein (200 μg) were precipitated with 4 volumes of acetone overnight at −20 °C. Pellets were washed and resuspended in 100 mM bicarbonate buffer (pH 8) for digestion with trypsin. Peptides were quantified (Pierce Quantitative Colorimetric Peptide Assay) and 10 μg of peptides per sample were taken for 16-plex tandem mass tag (TMT) labelling (Thermo Scientific) and pools were separated by a high pH reversed-phase peptide fractionation kit (Thermo Scientific) following the manufacturer’s instructions for in-house analysis by LC-MS/MS (Orbitrap Fusion Lumos MS, Thermo Scientific) as described (44). Differential abundance was determined using the Proteome Discoverer 3.1 software (Thermo Scientific) and *P*_adj_ < 0.1 was judged significant. Enrichment analysis for associated gene ontology terms of biologic processes (GO-BP) and Kyoto Encyclopedia of Genes and Genomes (KEGG) pathways was carried out using Metascape (https://metascape.org/) with default parameters and GO-BP clusters were manually annotated.

### Statistical analyses

Statistical analyses and graph construction were performed using Graphpad Prism 10 software. Numbers of animals or replicates and statistical tests used are described in the figure legends and *p* < 0.05 was judged significant and indicated in the figures as a single ∗ for differences between genotypes or ^$^ between conditions or time points. As no significant differences were seen between male and female mice, unless stated otherwise, data for both sexes were analyzed together to increase statistical power.

## Data availability

All relevant data are contained within the manuscript and associated Supporting Information. Raw data of LC-MS proteomics experiments can be shared upon request by contacting the corresponding author (manuel.johanns@uclouvain.be) or Dr Didier Vertommen (didier.vertommen@uclouvain.be).

## Supporting information

This article contains supporting information (Figs. S1 and S2, Table S1) as cited as such in the text.

## Conflict of interest

The authors declare that they have no conflicts of interest with the contents of this article.
