# Supplementary material for: Combined deletion of cytosolic 5′-nucleotidases IA and II lowers glycemia by improving skeletal muscle insulin action and lowering hepatic glucose production
Source: J Biol Chem. 2025 Feb 11;301(3):108295. doi: 10.1016/j.jbc.2025.108295 (PMC11938158; doi:10.1016/j.jbc.2025.108295)
Supplement: Supplementary Fig S1 [file mmc1.pdf]

Suppl. Figure S1

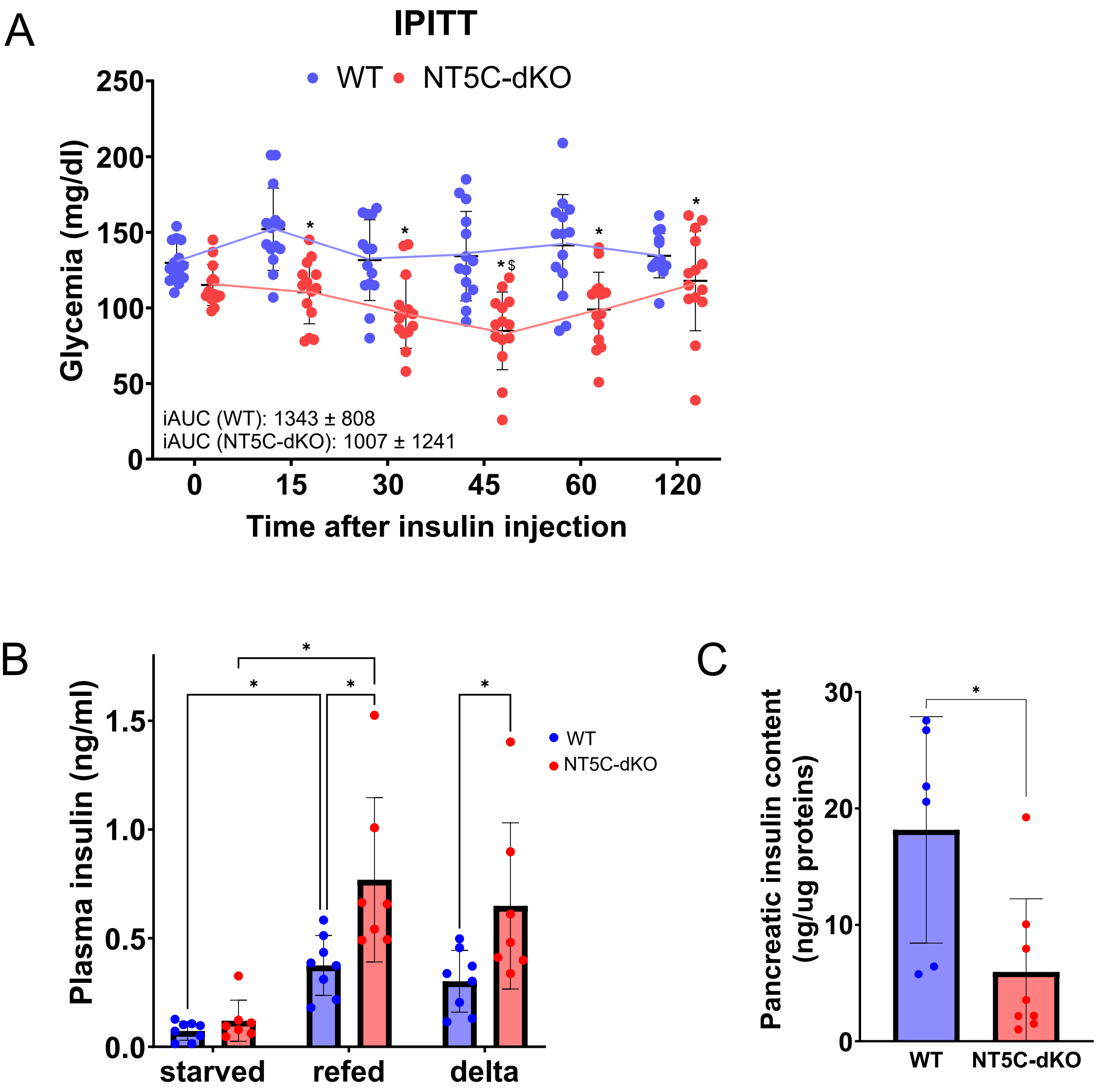

Supplemental Figure S1: Enhanced sensitivity, feeding-induced secretion but not pancreatic content of insulin i^{2}n NT5C-dKO mice. 4h-starved mice (N=14 per genotype, 7 males and 7 females) were subjected to an intraperitoneal insulin (0.1 U) tolerance test (IPITT) and glycemia was measured at indicated intervals (A); data were analyzed by 2-way ANOVA followed by Tukey's post-hoc test and * indicates a significant ($p<0.05$) difference between genotypes (compared to WT) and $^{\$}$ between timepoints compared to 0 min. iAUC is the incremental area under the curve using the starting value (0 min) of each series as baseline. Blood was harvested before and 30min after refeeding over-night starved mice (N=8 per genotype, 5 males and 3 females) for measurement of plasma insulin by ELISA (B); data were analyzed by 2-way ANOVA followed by Tukey's post-hoc test and * indicates a significant ($p<0.05$) difference between indicated conditions. "Delta" describes the difference in plasma insulin between the fasted and refed state for each animal. Pancreatic insulin content of over-night starved mice (N=6 per gentoype, 3 males and 3 females) was measured by ELISA in acid-ethanol extracts of whole pancreata (C); ; data were analyzed by an unpaired t-test with Welch's correction for unequal variances and * indicates a significant ($p<0.05$) difference. As no significant difference was seen between sexes, males anf females were analyzed together.
