# Supplementary material for: Combined deletion of cytosolic 5′-nucleotidases IA and II lowers glycemia by improving skeletal muscle insulin action and lowering hepatic glucose production
Source: J Biol Chem. 2025 Feb 11;301(3):108295. doi: 10.1016/j.jbc.2025.108295 (PMC11938158; doi:10.1016/j.jbc.2025.108295)
Supplement: Supplementary Fig S2 [file mmc2.pdf]

Suppl. Figure S2

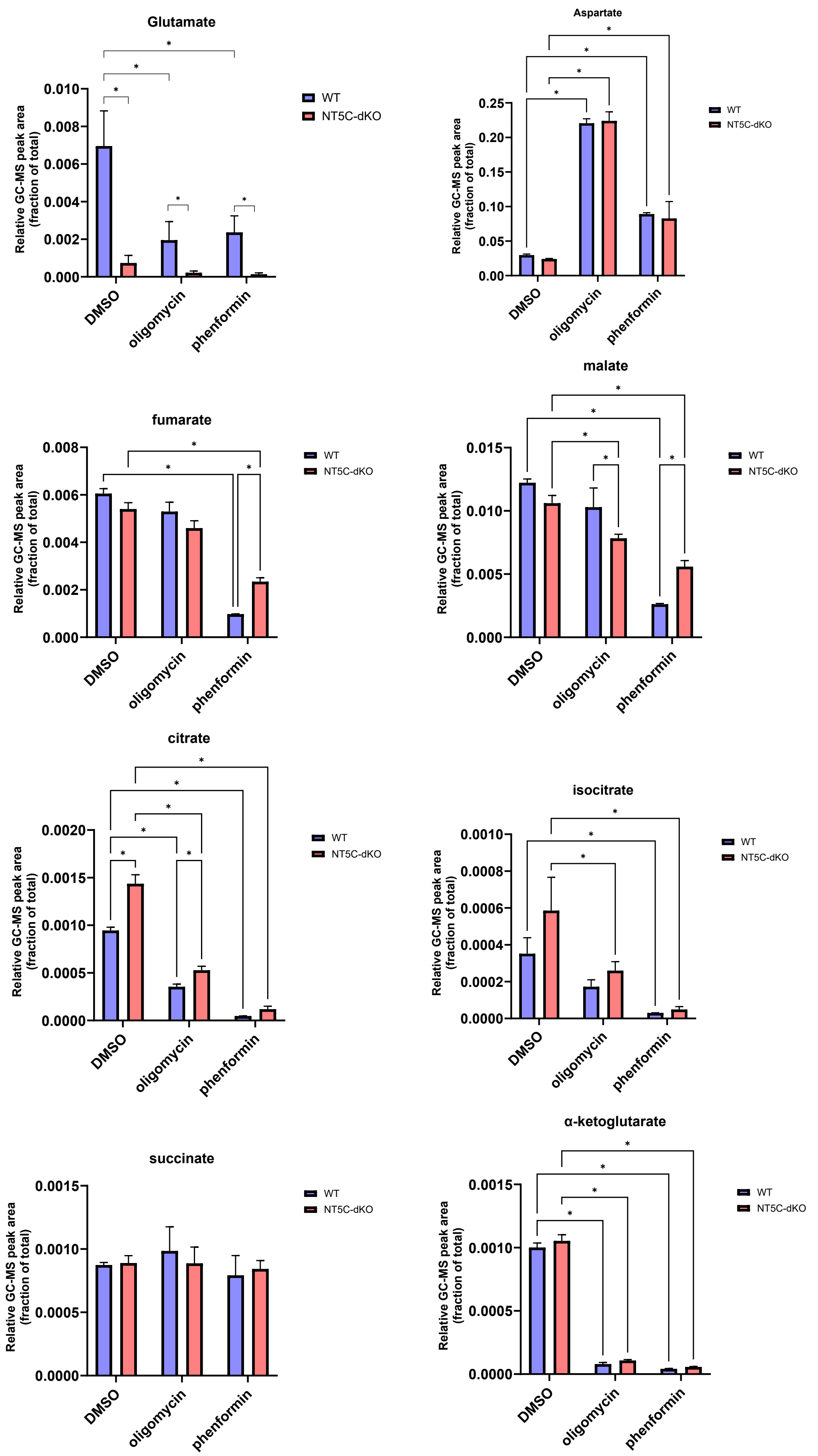

Supplemental Figure S2: Altered usage of TCA cycle intermediates and amino acids in NT5C-dKO mice. Over-night cultured hepatocytes from WT versus NT5C-dKO mice were incubated for 2 hours in normal media supplemented with DMSO (vehicle control), 1 uM oligomycin or 0.5 mM phenformin. Polar metabolites were extracted using methanol-chloroform and derivatized by methoxyamine and MSTFA for analysis by GC-MS. Data are means ± s.e.m. of 6 biological replicates and * indicates a significant ($p<0.05$) difference (2-way ANOVA with Tukey's post-hoc test).
